# The relationship between central obesity and bone mineral density: a Mendelian randomization study

**DOI:** 10.1186/s13098-022-00840-x

**Published:** 2022-05-03

**Authors:** Dengkui Du, Zhaopu Jing, Guangyang Zhang, Xiaoqian Dang, Ruiyu Liu, Jidong Song

**Affiliations:** 1 Department of Orthopaedics, The Second Affiliated Hospital, Xi’an Jiaotong University, No.157, Xiwu Road, Xi’an, 710004 Shaanxi Province China; 2 Department of Orthopaedics, Luoyang Central Hospital, Luoyang Central Hospital Affiliated to Zhengzhou University, Luoyang, 471009 Henan Province China

**Keywords:** Central obesity, Bone mineral density, Mendelian randomization

## Abstract

**Background:**

The relationship between obesity and osteoporosis is an important public health issue. The goal of this study was to investigate whether and to what extent central obesity traits affect bone mineral density (BMD).

**Methods:**

We conducted a two-sample Mendelian randomization analysis. Genomewide significant single nucleotide polymorphisms associated with waist circumference, hip circumference, waist-to-hip ratio, waist circumference adjusted by body mass index (WCadjBMI), hip circumference adjusted by BMI (HCadjBMI) and waist-to-hip ratio adjusted by BMI (WHRadjBMI) were obtained from a large-scale database containing 224,459 samples. The BMD summary dataset was obtained from a UK Biobank database including 265,627 participants.

**Results:**

The results provided strong evidence that the HCadjBMI trait was causally and negatively associated with BMD (*β*: − 0.135, 95% CI − 0.216 to − 0.054; *P* = 0.001), while the WHR trait was causally and positively associated with BMD (*β*: 0.194, 95% CI 0.062 to 0.325, *P* = 0.004). No significant effects were observed for other traits on BMD.

**Conclusions:**

This study indicates variations in the abilities of different central obesity traits to influence BMD. These results should be considered in further studies and public health measures on obesity and osteoporosis prevention strategies.

## Introduction

Osteoporosis is a systemic disease characterized by low bone mineral density (BMD), disruption of bone microarchitecture and an increased fracture risk [1]. With an aging population and expanding lifespan, osteoporosis is increasingly becoming a global epidemic. It is estimated that one in three women and one in five men over the age of 50 will experience osteoporotic fractures in their lifetime [2]. The prevalence of osteoporosis or low bone mass is expected to increase from 53 million to more than 70 million by 2030 in the USA [3]. BMD, the gold standard diagnostic criterion for osteoporosis [4], is closely related to obesity [5]. However, there are few data concerning the causal relationship between obesity and BMD, which is essential for the targeted prevention of osteoporosis.

General adiposity, represented by body mass index (BMI), is a common measure of obesity combining weight and height. However, BMI is an easily accessible but inaccurate indicator of obesity. Existing evidence suggests that central obesity, measured and reflected by body shape traits such as waist circumference (WC), hip circumference (HC), waist-to-hip ratio (WHR), WC adjusted by BMI (WCadjBMI), HC adjusted by BMI (HCadjBMI) and WHR adjusted by BMI (WHRadjBMI), might be more accurate than BMI when assessing obesity [6–8]. A genetic correlation has been found to be significant between central obesity and BMD [9]. Although there have been many observational studies with limited subjects probing the influence of central obesity on BMD, the reported results have been controversial [10, 11]. Additionally, these conventional studies cannot identify the causality between risk factors and disease.

Mendelian randomization (MR) analysis, a powerful method based on instrumental variables (IVs) to imitate the design of a randomized controlled trial, has been widely used in clinical causal inference. MR analysis is based on three IV assumptions: I, the IVs should be associated with the exposure; II, the IVs should influence the outcome only through the exposure (no horizontal pleiotropy); and III, the IVs should not be associated with confounders. A two‐sample MR estimates the causal effects based on the data where risk factors and outcomes are measured in different samples. To date, MR has not been applied to explore the causal effects of central obesity traits on BMD.

The aim of this study was to increase the understanding of the obesity-osteoporosis association by assessing the causal relationship between central obesity traits and BMD via a MR analysis using large genome-wide association study (GWAS) summary statistics.

## Methods

### Data sources

For the BMD dataset, the summary statistics for heel calcaneus BMD were obtained from UKB GWAS summary statistics including 265,627 participants of European descent [12].

For the central obesity dataset, the anthropometric summary statistic datasets for WC, WCadjBMI, HC, HCadjBMI, WHR, and WHRadjBMI from individuals included in the genetic investigation of anthropometric traits (GIANT) consortium (224,459 samples, European ancestry) were used [13]. Detail information for each trait is shown in Table 1. All datasets are available at the MR Base Database (http://www.mrbase.org/).

**Table 1:** Sample characteristics for traits in current study

Traits	Sample size	Independent SNPs number	Approximate variance Explained (%)
BMD	265,627	–^*^	–^*^
WC	224,459	45	1.1
WCadjBMI	245,746	63	1.3
HC	213,038	52	1.4
HCadjBMI	211,114	72	2.1
WHR	21,244	28	0.7
WHRadjBMI	224,452	36	0.1

^*^The independent SNPs number and variance explained by SNPs were different between each MR analysis

### SNP extraction and harmonization

The detailed method description is illustrated in our previous study [14]. In brief, significant genome-wide single nucleotide polymorph sins (SNPs) (*P* < 5 × 10^–8^) were extracted from the risk factor GWAS. To ensure that they were independent, the clumping method against a reference dataset of similar ancestry from the 1000 Genomes Project was used to prune SNPs for linkage disequilibrium (LD). Then, the summary statistics of SNPs associated with risk factors were extracted from the outcome datasets. If the SNPs were not included, LD proxies for the missing SNPs were estimated using the 1000 Genomes Project data. The LD proxies were limited to within 250 kb or 1000 SNPs with a minimum r^2^ = 0.6. Ambiguous SNPs with incompatible alleles (e.g., A/G vs. A/C) were excluded. Palindromic SNPs with ambiguous alleles (e.g., A/T vs. G/C), SNPs with incorrect effect alleles (e.g., G/T vs. T/G) and SNPs with strand issues (e.g., G/T vs. C/A) were harmonized by flipping the outcome variants.

### Two-sample Mendelian randomization analyses

First, we performed inverse variance weighted (IVW) analyses. This method analyzes each Wald ratio and provides a consistent estimate of the causal effect when all instrumental variables are valid. To detect and correct for pleiotropy in the IVW analyses, MR-Egger and weighted median analyses were then conducted. The slope from MR-Egger regression represents the causal effect estimate, and an intercept significantly different from zero (*P* < 0.05) is an indication of directional pleiotropy. The weighted median method gives unbiased estimates provided at least 50% of the information comes from nonpleiotropic SNPs.

The results were considered statistically significant at *P* < 0.05. All MR analyses were performed using the ‘TwoSampleMR’ package in R.

### Pleiotropy and sensitivity analyses

A key assumption of MR is no pleiotropy, but this is difficult to test directly. Therefore, we instead performed three sensitivity analyses. Directional pleiotropy was assessed based on the intercept from MR-Egger regression. A “leave-one-out” sensitivity analysis was performed to evaluate whether the analysis was biased by a single SNP that had a particularly large horizontal pleiotropic effect. Funnel plots were also used to assess the heterogeneity among SNPs.

## Results

### Studies included in the analysis

We selected three body shape anthropometric traits (WC, HC and WHR) and their BMI-adjusted traits (WCadjBMI, HCadjBMI and WHRadjBMI) as risk factors, and BMD was selected as the outcome in this study. Table 1 reports the independent, genome-wide significant SNPs for each trait, and how much of the phenotypic variation each set of SNPs explains.

### Causal effects of waist circumference on BMD

For the WC trait, one standard deviation (SD) higher of WC was associated with a 0.15 SD higher BMD according to IVW analysis (*β*: 0.150, 95% CI 0.073 to 0.227; *P* = 1.3 × 10^–4^). This association was consistent for the MR-Egger (*β*: 0.383, 95% CI 0.190 to 0.575; *P* = 3 × 10^–4^) and weighted median (*β*: 0.235, 95% CI 0.184 to 0.286; *P* = 1 × 10^–7^) methods. However, in the pleiotropy test, the MR-Egger result (intercept = − 0.007, *P* = 0.014) showed that genetic pleiotropy biased the WC-BMD results. Interestingly, as shown in the WCadjBMI trait, the positive causal associations in the WC-BMD analysis were completely counteracted after BMI adjustment. There was no evidence of directional pleiotropy in the MR-Egger regression analysis (intercept = − 0.001, *P* = 0.925).

### Causal effects of hip circumference on BMD

For the HC trait, the IVW analysis yielded no evidence to support a causal association between HC and BMD (*β*: 0.061, 95% CI − 0.019 to 0.141; *P* = 0.133). The MR-Egger and weighted median methods found a positive causal relationship in the HC-BMD results, but they were biased by directional pleiotropy (intercept = − 0.011, *P* = 0.003).

**Fig. 1:**
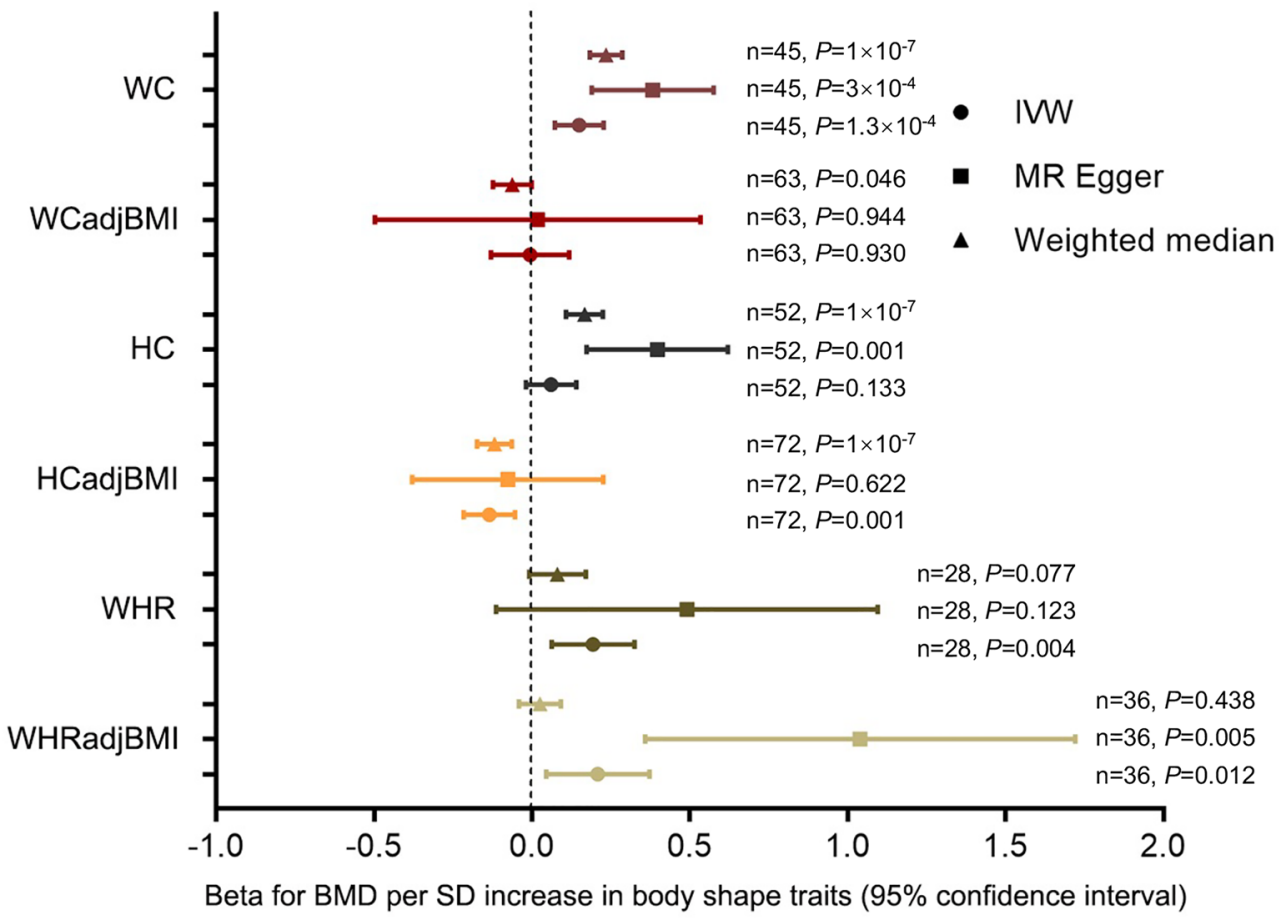
Effects of central obesity traits on BMD. Each point represents the combined causal estimate using all SNPs together in a single instrument with the IVW (**circle**), MR-Egger (**square**) and Weighted median (**triangle**) methods. Horizontal lines represent 95% confidence interval. n represents independent SNPs number and *P* represents *P*-value for each analysis

**Fig. 2:**
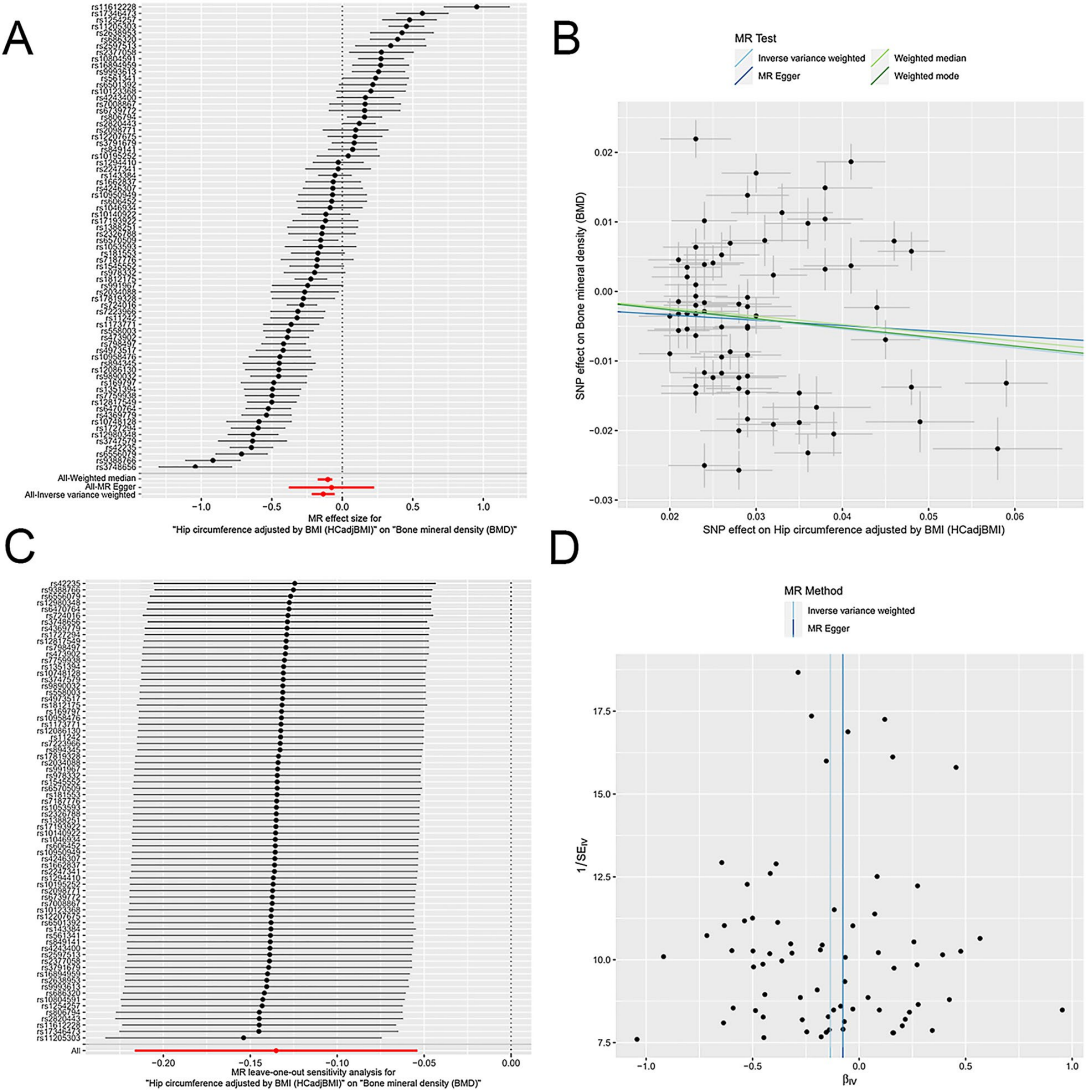
Effects of HCadjBMI on BMD. **A** Forrest plot of SNPs associated with HCadjBMI and BMD. **B** Scatter plot of SNPs associated with HCadjBMI and BMD. The slopes of each line represent the causal association for each method. **C** Leave-one-out analysis of SNPs associated with HCadjBMI and BMD. **D** Funnel plot of SNPs associated with HCadjBMI and BMD

However, after the HC trait was adjusted by BMI, HCadjBMI was negatively and causally associated with BMD, suggesting that an increase in HCadjBMI may cause a decrease in BMD. One SD higher of HCadjBMI was associated with a 0.135 SD lower BMD according to IVW analysis (*β*: − 0.135, 95% CI − 0.216 to − 0.054; *P* = 0.001). This causal relationship was consistent for the MR-Egger (*β*: − 0.076, 95% CI − 0.379 to 0.226, *P* = 0.622) and weighted median (*β*: − 0.118, 95% CI − 0.173 to − 0.063, *P* = 1 × 10^–7^) methods (Table 2, Fig. 1). Regarding pleiotropy and sensitivity, there was no evidence of directional pleiotropy in the MR-Egger regression analysis (intercept =  − 0.002, *P *  = 0.694). No single SNP strongly drove the overall effect of HCadjBMI on BMD in the leave-one-out analysis. The symmetry in the funnel plots also suggested that there were no violations of the IV assumptions (Fig. 2).

**Table 2:** The MR estimates of the causal effect of central obesity on BMD

Central obesity traits	IVW method			MR-Egger			Weighted median		
	Beta^*^	95% CI	*P*-value	Beta^*^	95% CI	*P*-value	Beta^*^	95% CI	*P*-value
WC	0.150	(0.073, 0.227)	1.3 × 10^− 4^	0.383	(0.190,0.575)	3 × 10^–4^	0.235	(0.184, 0.286)	1 × 10^–7^
WCadjBMI	− 0.005	(− 0.130, 0.119)	0.930	0.018	(− 0.497, 0.534)	0.944	− 0.062	(− 0.123,− 0.001)	0.046
HC	0.061	(− 0.019, 0.141)	0.133	0.397	(0.174, 0.621)	0.001	0.167	(0.109, 0.225)	1 × 10^–7^
HCadjBMI	− 0.135	(− 0.216, − 0.054)	0.001	− 0.076	(− 0.379, 0.226)	0.622	− 0.118	(− 0.173, − 0.063)	1 × 10^–7^
WHR	0.194	(0.062, 0.325)	0.004	0.491	(− 0.113, 1.096)	0.123	0.081	(− 0.009, 0.171)	0.077
WHRadjBMI	0.209	(0.046, 0.372)	0.012	1.040	(0.358, 1.721)	0.005	0.026	(− 0.040, 0.092)	0.438

^*^Beta: a ratio of changes in standard deviations of BMD to that of central obesity traits

### Causal effects of waist-to-hip ratio on BMD

**Fig. 3:**
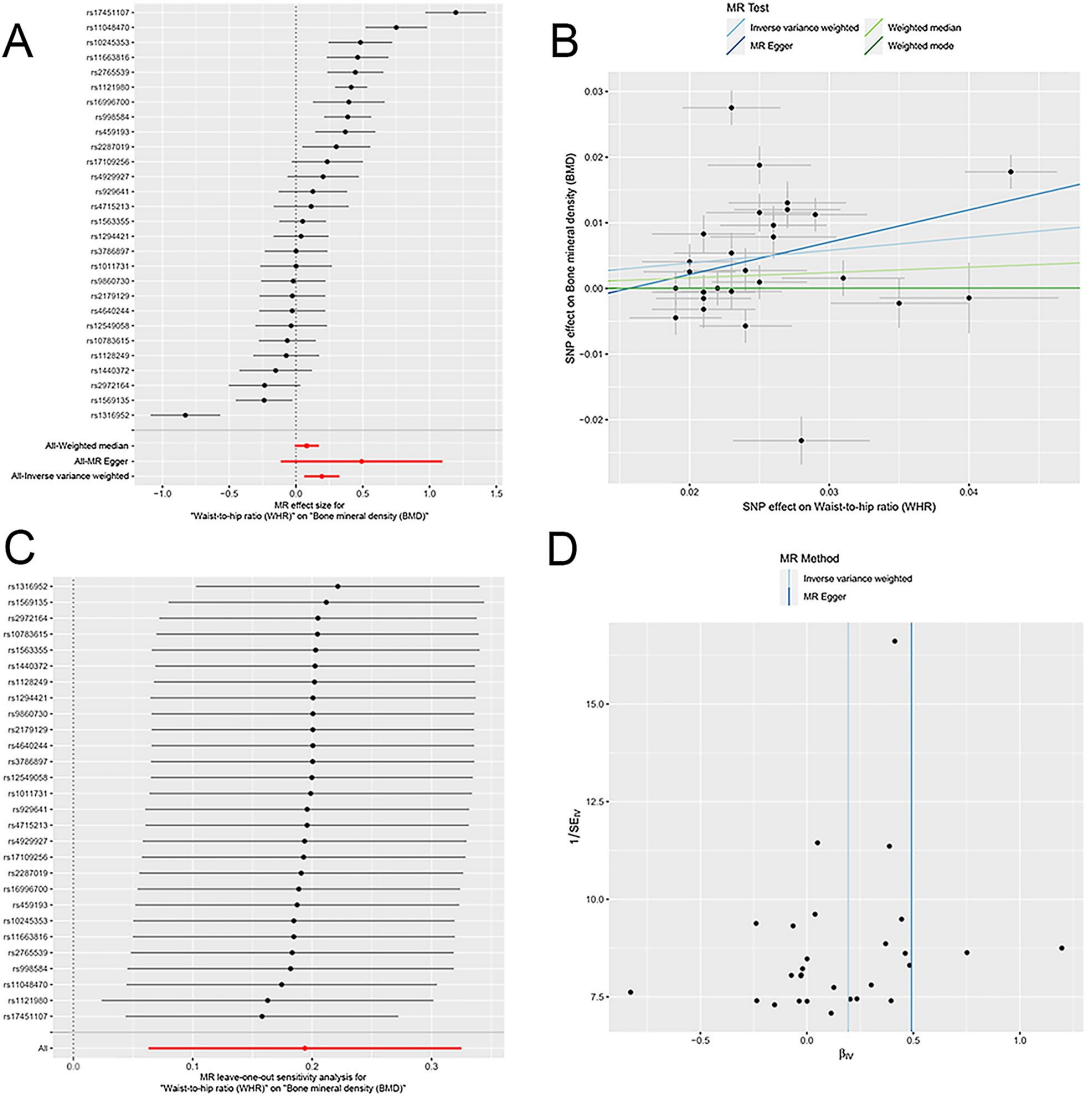
Effects of WHR on BMD. **A** Forrest plot of SNPs associated with WHR and BMD. **B** Scatter plot of SNPs associated with WHR and BMD. The slopes of each line represent the causal association for each method. **C** Leave-one-out analysis of SNPs associated with WHR and BMD. **D** Funnel plot of SNPs associated with WHR and BMD

We found the WHR trait was positively and causally associated with BMD, suggesting that an increase in WHR may cause an increase in BMD. One SD higher of WHR was associated with a 0.194 SD higher BMD according to IVW analysis (*β*: 0.194, 95% CI 0.062 to 0.325, *P* = 0.004). This causal relationship was consistent for the MR-Egger (*β*: 0.491, 95% CI − 0.113 to 1.096, *P* = 0.123) and weighted median (*β*: 0.081, 95% CI − 0.009 to 0.171, *P* = 0.077) methods (Table 2, Fig. 1). Their causal estimates were similar in direction and magnitude, and thus were unlikely to occur by chance alone. Regarding pleiotropy and sensitivity, there was no evidence of directional pleiotropy in the MR-Egger regression analysis (intercept = − 0.008, *P* = 0.332). The leave-one-out analysis and funnel plots also suggested that there were no violations of the IV assumptions (Fig. 3).

After the WHR trait was adjusted by BMI, WHRadjBMI was still positively and causally associated with BMD, as shown in the IVW (*β*: 0.209, 95% CI 0.046 to 0.372, *P* = 0.012) and MR-Egger (*β*: 1.040, 95% CI 0.358 to 1.721, *P* = 0.005) analyses. However, further analysis found that these associations may be biased by directional pleiotropy (Table 3, intercept = − 0.024, *P* = 0.019). Our further leave-one-out analysis found that a single-nucleotide polymorphism, rs7766106, had a particularly large horizontal pleiotropic effect and the MR estimate was mainly biased by it. Further phenome-wide association studies confirmed that the effects of rs7766106 on BMD was independent of its effects on WHRadjBMI.

**Table 3:** MR-Egger pleiotropy test of the causal effect of central obesity on BMD

Central obesity traits	MR-Egger method	
	Intercept	*P-*value
WC	− 0.007	0.014
WCadjBMI	− 0.001	0.925
HC	− 0.011	0.003
HCadjBMI	− 0.002	0.694
WHR	− 0.008	0.332
WHRadjBMI	− 0.024	0.019

## Discussion

This study aimed to explore whether and to what extent central obesity affects BMD. We performed a two-sample Mendelian randomization analysis to investigate the causal relationship between different central obesity measures and BMD. This study suggests there are variations in the ability of different central obesity traits to influence BMD. Our results provide genetic evidence that the HCadjBMI trait is causally and negatively associated with BMD, while the WHR trait is causally and positively associated with BMD. However, no significant effects were observed for WC, WCadjBMI, HC, and WHRadjBMI on BMD.

With the increasing prevalence of both obesity and osteoporosis in developed countries, whether obesity is a protective factor and which obesity trait is causally associated with BMD have been important public health issues. BMI, the most frequent index of general adiposity, is easily accessible and has clear categories [15]. Using large GWAS, our previous MR study assessed the association of overall obesity with BMD at various weight-bearing sites. We found that BMI was causally and positively associated with lumbar and heel calcaneus BMD but no statistically significant effects were observed for BMI on femoral neck or forearm BMD. This suggested that higher BMI played a causal role in increasing BMD and the genetic determination of BMI is different but similar across different skeleton [14]. BMI is an easily accessible index for overall obesity but it has several limitations. It is relatively unable to distinguish between lean mass and fat mass, and more importantly, between overall adiposity and abdominal adiposity [16]. Thus, the WHO is researching on central obesity indices to facilitate their appropriate use as an alternative or adjunct to BMI [17]. Additionally, existing evidence showed that central obesity is more strongly associated with chronic metabolic risk factors than overall obesity [8] and it may be underestimated in geriatric diseases including osteoporosis [18]. Therefore, apart from overall adiposity, understanding the central adiposity-osteoporosis relationship is an important part of obesity-osteoporosis studies. Our present study focuses on central adiposity and it is a continuation and supplement of our previous study on obesity-osteoporosis relationship. The similarity between the two studies is that they all focus on obesity-osteoporosis relationship and found differences in genetic BMD determination among various traits. The innovations are that central obesity indices compensate for deficiencies in overall obesity index and the present study classified risk factors into different indices and suggested there were differences in genetic BMD determination among various central obesity traits. Our findings might provide a reference for the levels at which central obesity measures predict the risk of osteoporosis.

When dealing specifically with central adiposity, the reported results from several observational studies are not consistent. In terms of WC, a cross-sectional study of 4663 Chinese adult men and another cross-sectional study of 3457 Chinese adults found that WC was a negative predictor of BMD [19, 20]. This relationship was also found in a Korean study of 8981 individuals [21] and a small study of 271 overweight adolescents [22]. However, positive associations were also revealed in several studies [23, 24]. Our study found a biased positive effect of WC on BMD but this was completely counteracted by BMI adjustment. In terms of HC, a cross-sectional study of 5287 subjects found that greater BMI and HC were associated with increased BMD at the lumbar spine and femoral neck [25]. Another small study including 60 women with polycystic ovary syndrome also found that BMD was positively correlated with BMI, WC and HC [26]. Both studies were conventional observational studies and could not distinguish the influence of BMI and HC. In our study, after adjusting for BMI, BMD was found to be negatively correlated with HCadjBMI, which indicates that abdominal deposition of fat measured by HC, independent of overall obesity, is associated with a lower BMD.

Moreover, by the advantages of distinguishing a particular intervention’s influence in MR analysis, we were able to find variations in the causal directions of different central obesity traits to influence BMD. In contrast to the HCadjBMI trait, we observed the protective effect of WHR, not WHRadjBMI, on BMD in this study. A Chinese cross-sectional study and another small study found that WHR was inversely related to bone mass [20, 27]. However, the results from another Chinese cohort study supported the protective effect of WHR on BMD [28].

The relationship between obesity and BMD is complex and complicated by many factors, because metabolic and endocrinological factors also interact with the biomechanical influence of the load on the bone determined by adipose tissue [29]. The cause-and-effect mechanisms between obesity and bone could come down to two main aspects, mechanical and metabolic. The increased mechanical loading and strain due to obesity have protective effects on bone modeling, density, and geometry [30], which may explain the higher BMD found in obese people. However, the loading factor and limitation of BMI concepts are not sufficient to explain all the actions of obesity on bone. The relative inability of BMI to distinguish between lean mass and fat mass makes it hard to distinguish whether BMI growth comes from muscle development or fat accumulation from overeating [31]. The WHR may be a better indicator of regional fat distribution, and a greater WHR indicates higher visceral fat and lower gluteal fat and muscle [32]. Metabolic factors related to obesity involving leptin and estrogens have protective effects on BMD [33]. Leptin, produced by adipocytes, may act peripherally by increasing osteoprotegerin levels leading to binding of RANKL, resulting in inhibition of osteoclastogenesis [34, 35]. Additionally, estradiol hormone therapy resulted in significant increase of BMD [36] and this may be because it can attenuate the subclinical inflammatory bone-microenvironment that is accompanied by an increase in oxidative stress and the generation of advanced glycation end products [37]. As a result, a high WHR might indicate that central obesity affects the levels of related factors to increase BMD, which may account for our results that WHR is causally and positively associated with BMD. The complex unmeasured confounders, small sample size, and interacting risk factors may account for the inconsistent results among conventional studies. Additionally, the obesity-osteoporosis relationship did not always translate into an obesity-fracture risk relationship. For example, a meta-analysis found that central obesity measured by WHR might be associated with an increased risk of hip fracture [38]. Nonbone factors including muscle function and soft-tissue thickness should also be considered when studying fracture risks [18].

This study has several important strengths. First, MR better accounts for the unmeasured confounders, and reverse causality biases existed in the observational studies. Second, MR uses genetic variants as IVs to imitate the design of randomized controlled trials (RCTs). It lies between observational studies and interventional trials and provides information about public health interventions in cases when an RCT may not be feasible. Third, the large sample size and robustly associated SNPs give sufficient power to detect causal effects with high precision.

The present study has several limitations. First, the data used in this study are from individuals of European descent. Further MR studies are needed in other populations because causality may depend on ethnicity. Second, it is impossible to explicitly test all three MR assumptions, especially assumption III. However, sensitivity analyses found no evidence of horizontal pleiotropy in several analyses. Third, we used GWAS data for heel eBMD instead of the current standard BMD because of the larger sample size. However, effect sizes were highly concordant between BMD and eBMD traits in terms of their genome-wide significant loci, indicating that their potential biological characteristics are similar [39]. The heel BMD traits were also successfully used in previous MR studies [40, 41]. Fourth, the analysis results reflected an individual’s lifetime exposure to risk factors and cannot reflect the effect of current treatment on prognosis.

## Conclusion

In conclusion, our Mendelian randomization study suggests variations in the ability of different central obesity traits to influence BMD. Our results provide strong evidence that HCadjBMI and WHR may be important factors causally influencing BMD. The HCadjBMI trait is causally and negatively associated with BMD, while the WHR trait is causally and positively associated with BMD. However, no significant effects were observed for WC, WCadjBMI, HC, and WHRadjBMI on BMD. These results should be considered in further studies and public health measures on obesity and osteoporosis prevention strategies.

## Data Availability

All data we used in the study was available at MR-Base platform.

## Abbreviations

BMD: Bone mineral density
BMI: Body mass index
WC: Waist circumference
HC: Hip circumference
WHR: Waist-to-hip ratio
WCadjBMI: WC adjusted by BMI
HCadjBMI: HC adjusted by BMI
WHRadjBMI: WHR adjusted by BMI
IVs: Instrumental variables
MR: Mendelian randomization
GWAS: Genome-wide association study
GIANT: Genetic investigation of anthropometric traits
SNPs: Single nucleotide polymorph sins
LD: Linkage disequilibrium
IVW: Inverse variance weighted
SD: Standard deviation
RCTs: Randomized controlled trials
